# Oleic acid attenuates asthma pathogenesis via Th1/Th2 immune cell modulation, TLR3/4-NF-κB-related inflammation suppression, and intrinsic apoptotic pathway induction

**DOI:** 10.3389/fimmu.2024.1429591

**Published:** 2024-10-03

**Authors:** Soon-Young Lee, Duc Dat Le, Chun-Sik Bae, Jin Woo Park, Mina Lee, Seung-Sik Cho, Dae-Hun Park

**Affiliations:** ^1^ College of Oriental Medicine, Dongshin University, Naju, Republic of Korea; ^2^ College of Pharmacy and Research Institute of Life and Pharmaceutical Sciences, Sunchon National University, Suncheon, Republic of Korea; ^3^ College of Veterinary Medicine, Chonnam National University, Gwangju, Republic of Korea; ^4^ Department of Biomedicine, Health & Life Convergence Sciences, BK21 Four, Biomedical and Healthcare Research Institute, Mokpo National University, Muan, Republic of Korea; ^5^ Department of Pharmacy, College of Pharmacy and Natural Medicine Research Institute, Mokpo National University, Muan, Republic of Korea

**Keywords:** oleic acid, asthma, immune balance, inflammation, apoptosis

## Abstract

WHO reported that asthma was responsible for 455,000 deaths in 2019 and asthma patients was evaluated 262 million in May 2023. The incidence is expected to increase as the average life expectancy increases, highlighting asthma as a significant health challenge in an aging society. The etiology of asthma is linked to an imbalance of Th1 and Th2 cells, respiratory inflammation, and pulmonary cell proliferation. The purpose of this study is to investigate the anti-asthmatic effect and potential mechanism of oleic acid. The anti-inflammatory effect of oleic acid was evaluated in an LPS-induced RAW 264.7 cell model, and immune modulation and the anti-apoptotic effect were measured in an ovalbumin-induced BALB/c mouse model. A variety of analytical procedures, such as MTT, qPCR, ELISA, Western blotting, immunofluorescence, gene transfection, immunohistochemistry, and several staining methods (Diff Quik, H&E, PAS), were used to evaluate the effectiveness and mechanisms of these methods. The results from *in vitro* experiments showed that oleic acid could reduce the levels of inflammatory cytokines (TNF-α, IL-6, and IL-1β), and molecular docking studies suggested that oleic acid could interact with TLR3 and TLR4 proteins to form ligand−protein complexes, showing good binding affinity. Additionally, oleic acid attenuated the expression of MAPK pathway components (JNK, p38 MAPK) and NF-κB pathway constituents (IκB, NF-κB, COX-2, PGE_2_). *In vivo* results indicated that oleic acid reduced the levels of inflammatory cells (WBCs and eosinophils) and IgE activity, reduced the expression of the Th2 cell transcription factor GATA-3, and decreased the levels of Th2/Th17-related cytokines (IL-4, TNF-α, and IL-6). Oleic acid also alleviated OVA-induced pathological changes in the lung, such as epithelial cell proliferation, inflammatory cell infiltration, and mucus hypersecretion. OVA restored apoptosis in lung epithelial cells by modulating the expression of Bcl-2 and Bax. In summary, oleic acid has potential as a novel candidate for asthma treatment through its ability to regulate immune cells, exert anti-inflammatory effects, and promote apoptosis, thereby ameliorating asthma manifestations.

## Graphical Abstract

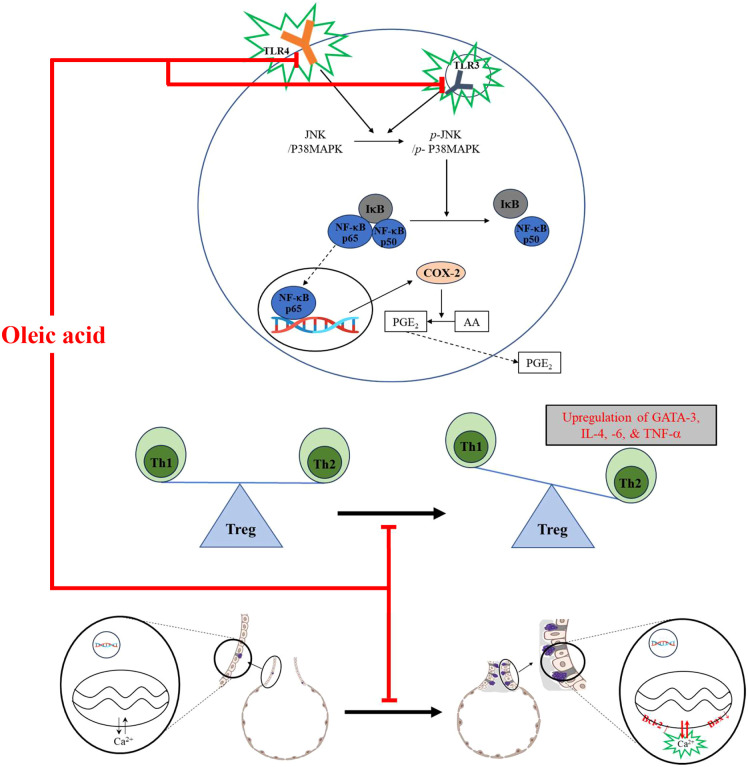

## Introduction

According to the World Health Organization Fact Sheet, the prevalence and severity of asthma are notably greater among children and elderly individuals than among adults. In 2019, asthma was responsible for 455,000 deaths, and as of May 2023, approximately 262 million individuals had been diagnosed with asthma (1). The incidence of asthma is expected to increase as the average life expectancy increases, highlighting asthma as a significant health challenge in an aging society (2).

Asthma is characterized as an incurable chronic inflammatory disease of the respiratory system (3). It presents with symptoms such as wheezing, coughing, tachypnea, and sleep apnea, which may be fatal (4). These symptoms are a manifestation of typical pathological lung alterations in asthma, such as airway remodeling, proliferation and metaplasia of lung epithelial cells, including goblet cells; mucus hypersecretion with submucosal hypertrophy; infiltration of inflammatory cells such as eosinophils and neutrophils; and contraction of airway smooth muscle (5). Asthma triggers are multifaceted and include allergens, environmental pollutants, cigarette smoke, cold weather, and genetic predispositions. These triggers are broadly categorized into indoor allergens (e.g., mites, cockroaches, pet dander) and outdoor allergens (e.g., mice, pollen, nuts, mold) (6, 7).

The pathogenesis of asthma is commonly attributed to inflammation in the lung airways (8), an imbalance in Th1 and Th2 cells (9), and the proliferation or metaplasia of various respiratory system cells (10). This disequilibrium arises from repeated exposure to allergens, leading to the upregulation of Th2 cell-associated factors (e.g., IL-4, IL-5, and IL-13) through the influence of Th17 cell-associated factors (e.g., TNF-α and IL-6) while concurrently downregulating Th1 cell-related cytokines (e.g., IL-12 and IFN-γ) (11–13). Asthma is a heterogeneous disease characterized by Th2-type airway inflammation with an influx of eosinophils and neutrophils. Oxidative stress exacerbates asthma by promoting airway inflammation and hyperresponsiveness, leading to respiratory system impairment, including airway remodeling and mucus hypersecretion (8).

Toll-like receptors (TLRs) play a pivotal role in innate and adaptive immunity, including inflammation (14). TLR4 stimulates NF-κB translocation as a transcription factor for the COX-2 protein via myeloid differentiation primary response protein 88 (MyD88) or Toll/IL-1 receptor domain-containing adaptor inducing IFN-β (TRIF) (15), and although the receptor proximal signaling of TLR3 and TLR4 differs, as TLR3 regulates the IL-1R pathway in NF-κB activation, similar to TLR4, TLR4 can also modulate NF-κB activation (16). The mitogen-activated protein kinase (MAPK) pathway, which functions as a mediator to deliver external stimuli to the inside of cells, is related to many biological processes in the cell system, including proliferation, survival, inflammation, and apoptosis (17). In particular, the activation of MAPK is strongly related to the NF-κB pathway (18). NF-κB is one of the pivotal regulators of inflammation (19); it regulates the levels of cyclooxygenase 2 (COX-2) and iNOS, thereby modulating prostaglandins to exacerbate inflammation (20).

Airway abnormalities, including hyperresponsiveness, are strongly related to the suppression of pulmonary epithelial cell death (10). Apoptosis, a form of programmed cell death, is crucial for various biological functions, including normal cell turnover, immune response regulation, and removal of damaged cells. Activation of jun N-terminal kinase (JNK) and p38 MAPK has been shown to promote apoptosis via the Bcl-2 family. In an ovalbumin-lipopolysaccharide (OVA-LPS)-induced animal model of asthma, bronchioalveolar epithelial cells in the lungs were found to undergo apoptosis through the activation of p38 MAPK, inhibition of Bcl-2, and induction of B-cell lymphoma 2 (Bcl-2)-associated X protein (Bax) (21).

The management of asthma, a complex disease, encompasses treatments primarily aimed at symptom relief. These treatments are categorized into relievers, controllers, and preventers. Relievers include short-acting β2-agonists and anticholinergics, controllers typically use long-acting β2-agonists, and preventers often consist of inhaled corticosteroids and leukotriene receptor antagonists. Dexamethasone is one of frequent used anti-asthmatic drug and anti-inflammation effect is caused by inhibition effect of NF-κB translocation from cytoplasm to nucleus (22) Moreover, combinations of preventers and controllers are frequently prescribed (23, 24). Despite the effectiveness of current asthma medications in relieving symptoms or reducing inflammation, a significant number of pediatric patients experience drug-related adverse effects, such as dry mouth, constipation, headache, nausea, tremors, nervous tension, muscle cramps, heart attacks, cataracts, glaucoma, peptic ulcers, pancreatitis, skin atrophy, hypertension, hyperlipidemia, immune suppression, and growth retardation (25–27). Consequently, there is a growing interest in developing safe and effective medications from natural sources that can minimize side effects.

Oleic acid, a monounsaturated fatty acid (MUFA), has been recognized for its anti-inflammatory properties and its ability to enhance immune function (28). Our recent studies have identified *Camellia japonica* oil, with oleic acid constituting 52% of its composition, as a contributor to the oil’s anti-asthmatic activity (29). Although the anti-asthmatic effects of oleic acid on *C. japonica* oil have been observed, the precise mechanisms by which oleic acid exerts its effects remain to be elucidated.

This study aimed to elucidate the anti-asthmatic mechanisms of oleic acid by investigating its interactions within the asthma pathway. Furthermore, we sought to establish oleic acid as a viable candidate for asthma treatment, emphasizing its potential effectiveness and safety.

## Materials and methods

### *In vitro* study

#### Cell culture

Mouse macrophage RAW 264.7 cells were obtained from the Korean Cell Line Bank (Seoul, Korea, Passage No. 23). These cells were cultured in Dulbecco’s modified Eagle’s medium (DMEM, Gibco, Paisley, UK) supplemented with 10% fetal bovine serum (FBS, Gibco) and 1% penicillin/streptomycin (Gibco) and maintained at 37°C in a 5% CO_2_ incubator (Thermo Fisher Scientific, Waltham, MA, USA).

#### Cell viability assay

To assess the cytotoxicity of oleic acid (Sigma−Aldrich, St. Louis, MO, USA) and establish the maximum nontoxic dose for treatment, an MTT (3-(4,5-dimethylthiazol-2-yl)-2,5-diphenyltetrazolium bromide) assay was conducted. RAW 264.7 cells were plated at 1 × 10^4^ cells/well in 96-well plates and incubated at 37°C in a 5% CO_2_ atmosphere for 12 h. Subsequently, the cells were treated with eight concentrations of oleic acid, ranging up to 100 μM, in six replicates per dose. This procedure was performed in triplicate. Following a 24-h treatment period, a 5 mg/mL MTT solution (Sigma−Aldrich) was added to each well and allowed to incubate for 2 h. The medium was then removed, and 100 μL of dimethyl sulfoxide (DMSO) was added to solubilize the formazan crystals. The absorbance at 570 nm was measured using a Multiskan SkyHigh spectrophotometer (Thermo Fisher Scientific, Waltham, MA, USA). To evaluate the anti-inflammatory effects of oleic acid, a lipopolysaccharide (LPS, Sigma−Aldrich)-induced cell model was employed, with a protocol mirroring that of the cytotoxicity assessment, but utilizing a medium containing LPS for oleic acid treatment. The results were recorded using a Multiskan SkyHigh instrument.

#### Nitric oxide assay

Nitric oxide (NO) levels were quantified using the supernatant from the cell culture medium. RAW 264.7 cells were seeded at a density of 2 × 10^5^ cells/well in 24-well plates and incubated for 12 h in a CO_2_ incubator. The cells were treated with five concentrations (0, 5, 10, 25, or 50 μM) of oleic acid in triplicate for 2 h. Following the addition of 10 ng/mL LPS, the cells were incubated for another 24 h. Afterward, the medium was harvested from all the wells. For NO quantification, 50 μL of a standard sodium nitrite solution (Daejung Chemicals & Metals, Gyeonggi, Korea) or the respective oleic acid concentrations were dispensed into a 96-well plate. Subsequently, 50 μL of Griess reagent (Sigma−Aldrich) was added to each well. After incubating for 15 min, the absorbance was measured at 540 nm using a Multiskan SkyHigh spectrophotometer.

#### Real-time polymerase chain reaction analysis

RT−PCR was used to quantify changes in the expression of genes such as TNF−α and IL−6 in cells. RAW 264.7 cells (6 × 10^5^ cells/well) were plated in 6-well plates and incubated in a CO_2_ incubator for 12 h. Following 2 h of oleic acid treatment, 10 ng/mL LPS was added, and the cells were incubated for an additional 24 h. Total RNA was extracted from the cells using TRIzol reagent (Thermo Fisher Scientific). The RT−PCR mixture included SB-Green qRT−PCR one-step master mix (LeGene Biosciences, San Diego, CA, USA) and primers. The RT−PCR cycling conditions were as follows: cDNA synthesis at 50°C for 10 min, denaturation at 95°C for 5 min, and 40 cycles of annealing/extension at 65°C for 30 s. The data were acquired using the q-Tower2.2 system (Analytik Jena GmbH, Jena, Germany), and the sequences of the forward and reverse primers used are listed in **Table 1**.

**Table 1:** The gene sequences used in the *in vitro* study.

Gene	Sequence	
TNF-α	Forward	5’-CTGAGTTCTGCAAAGGGAGAG-3’
	Reverse	5’-CCTCAGGGAAGAATCTGGAAAG-3’
IL-6	Forward	5’-GATAAGCTGGAGTCACAGAAGG-3’
	Reverse	5’-TTGCCGAGTAGATCTCAAAGTG-3’
GAPDH	Forward	5’-GTGGAGTCATACTGAACATGTAG-3’
	Reverse	5’-AATGGTGAAGGTCGGTGTG-3’

#### Enzyme-linked immunosorbent assay analysis

ELISA was conducted to measure the concentrations of inflammatory mediators such as TNF-α and IL-1β. The medium supernatant, which was stored in a deep freezer (NIHON FREEZER, Tokyo, Japan), was used for this purpose. ELISA kits for TNF-α and IL-1β were obtained from BD Biosciences (San Jose, CA, USA) and Invitrogen (Carlsbad, CA, USA), respectively. The assays were performed according to the manufacturers’ protocols. The results were read using the Multiskan SkyHigh device.

#### Western blot analysis

RAW 264.7 cells were seeded at densities of 4 × 10^6^ or 2 × 10^6^ in 100φ dishes and incubated for 12 h. The cells were treated with oleic acid for 2 h, followed by 10 ng/mL LPS for either 30 min or 24 h. Cell lysates were prepared using RIPA-based lysis buffer supplemented with a protease inhibitor cocktail (Thermo Fisher Scientific). The lysates were centrifuged at 13,000 rpm for 20 min at 4°C, the supernatant was collected, and the protein concentration was determined using the Bradford assay. Proteins were denatured by heating with SDS−PAGE loading buffer at 95°C for 5 min. Following SDS−PAGE, proteins were transferred onto PVDF membranes (Microlab Scientific, KLN, HK). Primary antibodies against *p*-JNK (9255S), JNK (9252S), *p*-p38 MAPK (4511S), p38 MAPK (9212S), *p*-NF-κB (MA5-15160), NF-κB (51–0500), *p*-IκB (MA5-15224), IκB (MA5-16152), COX-2 (PA1-9032), PGE_2_ (bs-2639R), iNOS (PA3-030A), and GAPDH (MA5-15738) (all from Cell Signaling Technology, Danvers, MA, USA, or Invitrogen, Carlsbad, CA, USA, or Bioss, Woburn, MA, USA) were applied to 5% skim milk. The membranes were washed with 0.1% Tween-20 Tris-buffered saline and incubated with secondary antibodies (goat anti-rabbit IgG (111-035-003) or goat anti-mouse IgG (115-035-003), both from Jackson ImmunoResearch, West Grove, PA, USA). Bands were visualized using an enhanced chemiluminescence (ECL) reagent (Thermo Fisher Scientific) and imaged using the Davinch-Western™ Imaging System (Davinch-K, Seoul, Korea). Protein expression relative to that of GAPDH was analyzed using ImageJ software (National Institutes of Health and the Laboratory for Optical and Computational Instrumentation, USA).

#### Immunofluorescence analysis

To verify the activity of the NF-κB/COX-2 pathway, immunofluorescence analysis was conducted. RAW 264.7 cells (3 × 10^5^ cells/well) were seeded into a 4-well chamber slide. Following the same pretreatment procedure used for Western blotting, the cells were treated with oleic acid for 30 min. Primary antibodies against NF-κB (Invitrogen, 51-0500) and COX-2 (Invitrogen, PA1-9032) were applied for 16 h. Subsequently, the cells were incubated with Alexa Fluor 488-conjugated anti-rabbit IgG (Invitrogen, A32731) or Alexa Fluor 555-conjugated anti-goat IgG (Invitrogen, A32816) for 2 h. For nuclear counterstaining, 4′,6-diamidino-2-phenylindole dihydrochloride (DAPI, Thermo Fisher Scientific) was used. Imaging was performed using a K1-Fluo confocal microscope (Nanoscope Systems, Daejeon, Korea).

#### Gene transfection

Gene transfection was carried out to examine changes in the expression of genes such as TNF-α, IL-6, and IL-1β following intracellular TLR4 knockdown. RAW 264.7 cells were seeded in 6-well plates (4 × 10^5^ cells/well) or 24-well plates (1 × 10^5^ cells/well) with antibiotic-free culture medium and incubated in a CO_2_ incubator for 12 h. siRNA and Lipofectamine RNAiMAX (Invitrogen) were diluted separately, mixed, incubated at room temperature for 10 min, and then added to the cells. The culture medium was replaced 6 h after transfection, and the cells were cultured for an additional 18 h. After treatment with various concentrations of oleic acid (0, 5, 10, 25, or 50 μM) for 2 h, 10 ng/mL LPS was added, and the cells were cultured for 24 h. Two days after transfection, the cells were harvested for ELISA and RT−PCR analysis.

### *In vivo* study

#### Ethics statement

The animal experiments were approved by the Institutional Animal Care and Use Committee of Chonnam National University (CNU IACUC-YB-2018-35).

#### Animal experiments

Forty-eight BALB/c mice (female, 16 g ~ 20 g, Samtako, Osan, Korea) were divided into six groups: control (CON), ovalbumin-treated for asthma induction (OVA), dexamethasone (1 mg/kg/d; SAMNAM Pharm, Chungcheongnam-do, Korea) post ovalbumin treatment (DEX), and three different dosages of oleic acid (50, 125, and 250 mg/kg/day). The maximum dose was determined to be an anti-asthma drug. All mice, except those in the CON group, received intraperitoneal injections of 20 μg of ovalbumin and 1 mg of aluminum hydroxide hydrate (Sigma−Aldrich) in 500 μL of saline on days 1 and 8. From days 21 to 25, the animals were orally administered dexamethasone (1 mg/kg/day) and oleic acid (50, 125, and 250 mg/kg/day) and subjected to inhalation of 5% ovalbumin using a nebulizer (3 mL/min, NE-U17, OMRON, Kyoto, Japan) for 30 min to induce asthma.

#### Bronchoalveolar liquid fluid analysis

Following the last treatment, all animals were anesthetized with an intraperitoneal injection of 50 mg/kg Zoletil (Virbac, Fort Worth, TX, USA). During deep anesthesia, a flexible feeding needle was inserted through the trachea of four animals from each group to collect BALF. The lungs of the remaining four animals in each group were harvested. The BALF was collected with 0.4 mL of phosphate-buffered saline (PBS) and centrifuged for 5 min at 3,000 rpm at 4°C using an M15R microcentrifuge (Hanil Scientific). Differential cell counts were performed on the resuspended BALF white blood cells (WBCs) using a Hemavet Multispecies Hematology System (Drew Scientific, Waterbury, CT, USA) and the Diff Quik staining set (Thermo Fisher Scientific).

#### Immunoglobulin E in serum analysis

Serum samples for IgE analysis were prepared by centrifuging the blood at 12,000 rpm for 15 min. The IgE levels were measured using an OptEIA Mouse ELISA Kit (BD Biosciences), and the results were read with a Multiskan SkyHigh device.

#### Histopathological observation

The anti-asthmatic effects of oleic acid were assessed by examining pathological changes via hematoxylin and eosin (H&E) and periodic acid–Schiff (PAS) staining. Lung tissues were fixed in 10% (v/v) formaldehyde for several days, dehydrated through graded ethanol solutions from 99.9% to 70%, and embedded in paraffin. Sections were cut longitudinally from the paraffin-embedded lung tissues and stained with hematoxylin (Vector Laboratories, Newark, CA, USA) and eosin (Sigma−Aldrich) to observe morphological alterations. PAS staining was performed using periodic acid (Sigma−Aldrich) and Schiff’s reagent (Merck Millipore, Darmstadt, Germany) to detect changes in bronchoalveolar mucus secretion.

#### RT−PCR analysis

RT−PCR was used to measure changes in the gene expression levels of IL-4, TNF-α, and IL-6 in lung tissue. The total RNA extraction and RT−PCR procedures were consistent with those described in the “*in vitro* study” subsection. The sequences for the forward and reverse primers are detailed in **Table 2**.

**Table 2:** The gene sequences used in the *in vivo* study.

Gene	Sequence	
TNF-α	Forward	5’-CTGAGTTCTGCAAAGGGAGAG-3’
	Reverse	5’-CCTCAGGGAAGAATCTGGAAAG-3’
IL-4	Forward	5’-ACAGGAGAAGGGACGCCAT-3’
	Reverse	5’-GAAGCCCTACAGACGAGCTCA-3’
IL-6	Forward	5’-GATAAGCTGGAGTCACAGAAGG-3’
	Reverse	5’-TTGCCGAGTAGATCTCAAAGTG-3’
GAPDH	Forward	5’-GTGGAGTCATACTGAACATGTAG-3’
	Reverse	5’-AATGGTGAAGGTCGGTGTG-3’

#### ELISA analysis

To determine the levels of IL-4, TNF-α, and IL-6 in lung tissue, an OptEIA Mouse ELISA Kit (BD Biosciences) was used following the manufacturer’s protocol. Tissue samples were homogenized in lysis buffer containing a protease inhibitor cocktail and RIPA buffer (Thermo Fisher Scientific). After homogenization, centrifugation was carried out at 12,000 rpm for 15 min, and the supernatant was used for subsequent analyses. The measurements were conducted using a Multiskan SkyHigh device.

#### Immunofluorescence analysis

Lung tissue sections were dehydrated using ethanol. The antigen retrieval process was conducted for 40 min, followed by incubation with primary antibodies diluted in 1% bovine serum albumin (BSA), including GATA-3 (OriGene, Rockville, MD, USA, TA305795), *p*-NF-κB (Invitrogen, MA5-15160), COX-2 (Invitrogen, PA1-9032), Bcl-2 (Abcam, Cambridge, UK, ab59348), and Bax (Invitrogen, MA-514003), for 1 h. Secondary antibodies and nuclear staining were carried out using the same reagents as in the *in vitro* study. The terminal deoxynucleotidyl transferase dUTP nick end labeling (TUNEL) assay was conducted using the Click-iT™ Plus TUNEL Assay (Invitrogen), with imaging performed on a K1-Fluo confocal microscope.

#### Immunohistochemical analysis

Immunohistochemistry was employed to evaluate changes in prostaglandin E_2_ (PGE_2_) levels. Sections (5 μm thick) were cut from paraffin-embedded lung tissue blocks and deparaffinized. An ImmPRESS Universal Polymer Kit (Vector Laboratories) was used for immunohistochemical analysis. Antigen retrieval was performed, followed by the application of the blocking serum from the kit to prevent nonspecific binding. The primary antibody targeting PGE_2_ (Bioss, bs-2639R) was applied for 1 h and then incubated with a biotinylated panspecific antibody for 30 min. Color development was achieved using 3,3′-diaminobenzidine (DAB) substrate (Vector Laboratories), and counterstaining was performed with hematoxylin. Imaging was acquired with an Axioscope A1 microscope.

### *In silico* study

Three-dimensional structures of the TLR3 (PDB ID: 1ZIW) and TLR4-MD2 (PDB ID: 5IJD) proteins were obtained from the RCSB Protein Data Bank. The structure of oleic acid was obtained from PubChem in SDF format and converted to PDBQT format using Open Babel. Protein and ligand preparations were executed using MGL Tools 1.5.6. The grid box dimensions were set using PyMOL or based on previous reports (30–32). A total of 100 runs were performed with default parameters using the Lamarckian genetic algorithm. Ligand−protein interactions were analyzed using AutoDock 4.2. Visualizations were created using PyMOL and Discovery Studio 2021 tools.

### Statistics

The data are presented as the mean ± standard deviation (SD). Differences between groups were assessed using one-way ANOVA, followed by Dunnett’s multiple comparison test to confirm specific differences. The significance threshold was set at *p* < 0.05.

## Results

### *In vitro* study

#### Safety and anti-inflammatory/oxidative effects of oleic acid in RAW 264.7 cells

**Figure 1:**
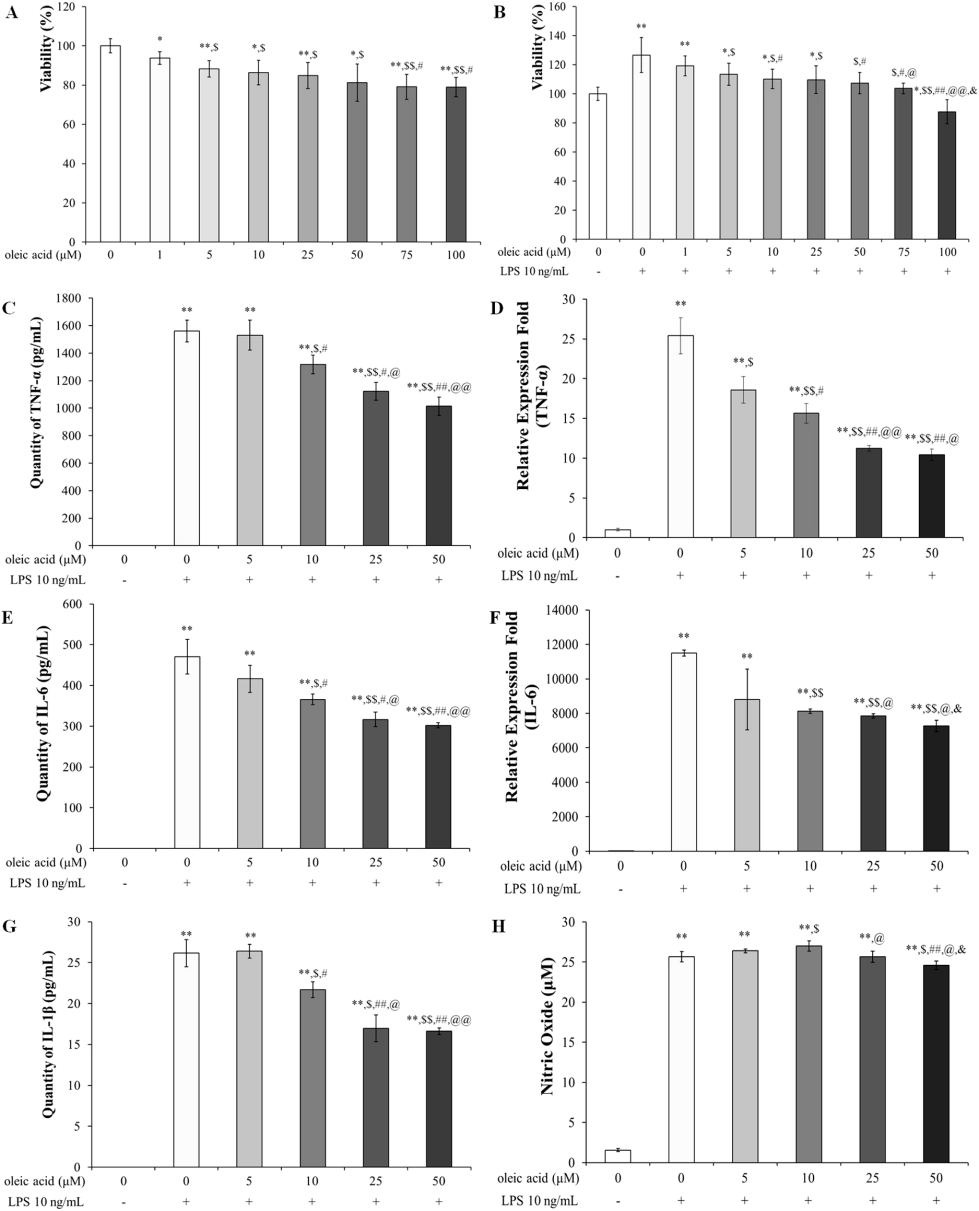
Safety of oleic acid in RAW 264.7 cells and effect of oleic acid on inflammatory cytokines. **(A)** Cell viability was reduced in a dose-dependent manner by oleic acid treatment up to 100 μM; notably, 50 μM oleic acid treatment maintained approximately 80% cell viability. ^*^ *p* < 0.05 vs. 0 μM oleic acid treatment; ^**^ p < 0.001 vs. 0 μM oleic acid treatment; ^$^ *p* < 0.05 vs. 1 μM oleic acid treatment; ^$$^ *p* < 0.001 vs. 1 μM oleic acid treatment; ^#^ *p* < 0.05 vs. 5 μM oleic acid treatment. **(B)** Compared with the control treatment, LPS treatment increased cell viability to 130%, whereas oleic acid treatment decreased viability in a dose-dependent manner (n = 6). ^*^ *p* < 0.05 vs. 0 μM oleic acid; ^**^ p < 0.001 vs. 0 μM oleic acid; ^$^ *p* < 0.05 vs. 10 ng/mL LPS; ^$$^ *p* < 0.001 vs. 10 ng/mL LPS; ^#^ *p* < 0.05 vs. 1 μM oleic acid; ^##^ *p* < 0.001 vs. 1 μM oleic acid; ^@^ *p* < 0.05 vs. 5 μM oleic acid; ^@@^ *p* < 0.001 vs. 5 μM oleic acid; ^&^ *p* < 0.05 vs. 10 μM oleic acid. Oleic acid decreased the expression level of LPS-induced cytokines in RAW 264.7 cells in a concentration-dependent manner (n = 5). Cells were treated with 0-50 μM oleic acid for 2 h and then exposed to 10 ng/mL LPS for 24 h. **(C)** TNF-α levels were measured by ELISA. **(D)** TNF-α quantified by RT−PCR. **(E)** IL-6 levels were assessed by ELISA. **(F)** IL-6 levels were determined by RT−PCR. **(G)** IL-1β levels were analyzed by ELISA. **(H)** In RAW 264.7 cells, treatment with 25 μM or 50 μM oleic acid reduced NO production. ^**^ p < 0.001 vs. 0 μM oleic acid treatment; ^$^ *p* < 0.05 vs. 10 ng/mL LPS treatment; ^$$^ *p* < 0.001 vs. 10 ng/mL LPS treatment; ^#^ *p* < 0.05 vs. 5 μM oleic acid treatment; ^##^ *p* < 0.001 vs. 5 μM oleic acid treatment; ^@^ *p* < 0.05 vs. 10 μM oleic acid treatment; ^@@^ *p* < 0.001 vs. 10 μM oleic acid treatment; ^&^ *p* < 0.05 vs. 25 μM oleic acid treatment. The results are expressed as the mean ± standard deviation.

The safety range and anti-inflammatory effects of oleic acid on RAW 264.7 cells were assessed using an MTT assay at concentrations ranging from 0 μM to 100 μM. At 100 μM, approximately 80% of the cells were oleic acid-treated (**Figure 1A**), and oleic acid suppressed approximately 90% of the LPS-induced cell proliferation (**Figure 1B**). Although oleic acid was shown to be safe and exhibited immune-suppressive effects up to 100 μM, subsequent studies were conducted with a maximum concentration of 50 μM to ensure safety. Oleic acid significantly decreased the release of proinflammatory cytokines such as TNF-α, IL-6, and IL-1β in a dose-dependent manner. ELISA revealed that the levels of these cytokines were significantly elevated in the LPS-treated group relative to those in the control group and were reduced by approximately 30% with 50 μM oleic acid treatment (**Figures 1C, E, G**). RT−PCR analyses indicated that the levels of TNF-α and IL-6 were significantly greater in the LPS-treated group than in the control group, and 50 μM oleic acid decreased TNF-α levels by approximately 60% and IL-6 levels by approximately 30% (**Figures 1D, F**). Treatment with oleic acid at concentrations up to 10 μM slightly increased NO levels, but 50 μM oleic acid suppressed NO production (**Figure 1H**).

#### Oleic acid inactivates the NF-κB signaling pathway via MAPK signaling

**Figure 2:**
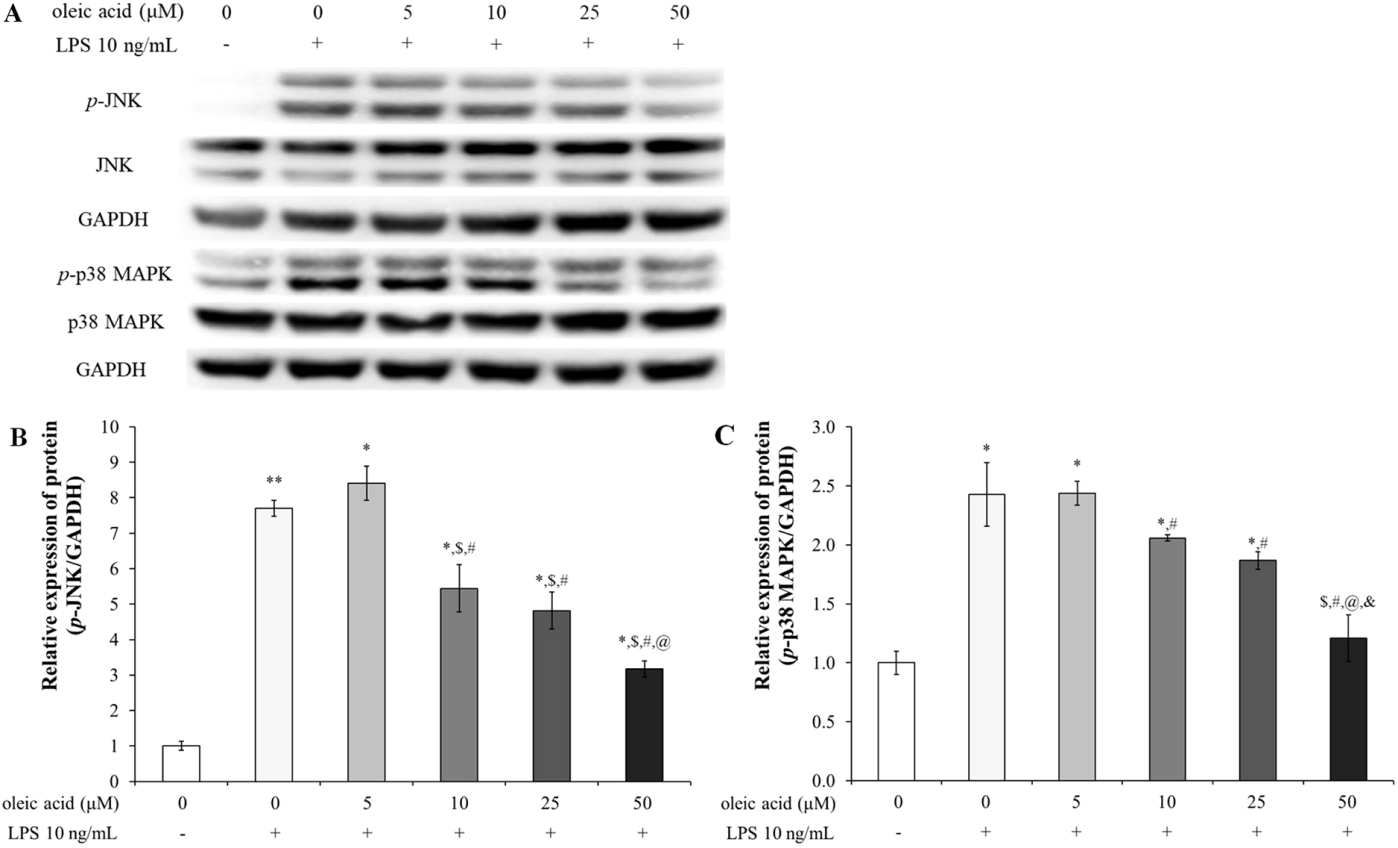
Effect of oleic acid on the MAPK signaling pathway. **(A)** Oleic acid reduced LPS-induced inflammation by inhibiting the activation of the JNK and p38 MAPK signaling pathways in RAW 264.7 cells (n = 4). Cells were treated with 0–50 μM oleic acid for 2 h and then exposed to 10 ng/mL LPS for 30 min. Phosphorylated JNK **(B)** and *p*-p38 MAPK **(C)** protein levels, which were normalized to those of GAPDH, decreased. ^*^ p < 0.05 vs. 0 μM oleic acid treatment; ^**^ p < 0.001 vs. 0 μM oleic acid treatment; ^$^ *p* < 0.05 vs. 10 ng/mL LPS treatment; ^#^ *p* < 0.05 vs. 5 μM oleic acid treatment; ^@^ *p* < 0.05 vs. 10 μM oleic acid treatment; *^&^ p* < 0.05 vs. 25 μM oleic acid treatment. The results are expressed as the mean ± standard deviation.

Oleic acid treatment decreased the protein expression levels of *p*-JNK and *p*-p38 MAPK (**Figure 2**). In the group treated with 10 ng/mL LPS, the relative expression levels of *p*-JNK and phosphorylated p38 MAPK (*p*-p38 MAPK) were significantly greater than those in the control group. The expression level of *p*-JNK slightly increased with 5 μM oleic acid but decreased in a concentration-dependent manner beginning at 10 μM oleic acid (**Figure 2B**). Furthermore, the expression level of *p*-p38 MAPK was reduced in a concentration-dependent manner across the oleic acid-treated groups (**Figure 2C**).

**Figure 3:**
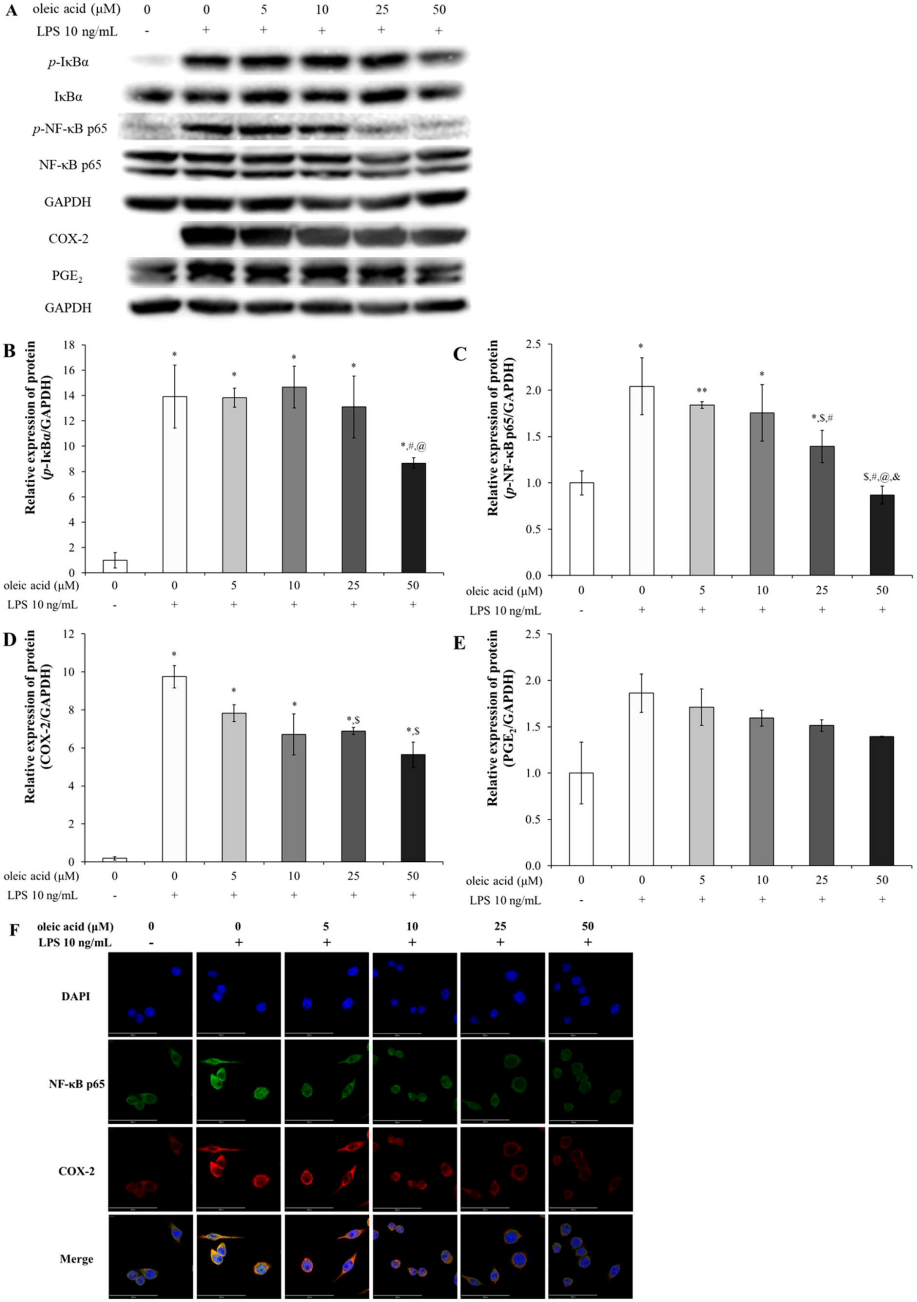
Effect of oleic acid on the NF-κB signaling pathway. **(A)** Oleic acid attenuated LPS-induced inflammation by suppressing the NF-κB/COX-2/PGE_2_ pathway in RAW 264.7 cells (n = 4). After treatment with 0–50 μM oleic acid for 2 h, the cells were treated with 10 ng/mL LPS for either 30 min or 24 h. **(B–E)** Phosphorylated protein levels were normalized to those of GAPDH. **(F)** Immunofluorescence revealed that LPS treatment enhanced the translocation of NF-κB p65 (green fluorescence) to the nucleus and upregulated COX-2 (red fluorescence) in the cytoplasm, whereas oleic acid dose-dependently reduced both COX-2 expression and NF-κB translocation. ^*^ p < 0.05 vs. 0 μM oleic acid treatment; ^**^ p < 0.001 vs. 0 μM oleic acid treatment; ^$^ *p* < 0.05 vs. 10 ng/mL LPS treatment; ^#^ *p* < 0.05 vs. 5 μM oleic acid treatment; ^@^ *p* < 0.05 vs. 10 μM oleic acid treatment; ^&^ *p* < 0.05 vs. 25 μM oleic acid treatment. The results are expressed as the mean ± standard deviation. Scale bar, 50 μm; magnification, 1000×.

In the control group of RAW 264.7 cells without LPS treatment, the NF-κB signaling pathway was inactive but became active upon LPS treatment (**Figure 3**). Consequently, the expression levels of *p*-IκB, *p*-NF-κB p65, COX-2, and PGE_2_ were significantly upregulated in the LPS-treated group. In contrast, groups treated with varying concentrations of oleic acid exhibited a concentration-dependent decrease in the expression of these proteins, mitigating LPS-induced inflammation via the NF-κB pathway (**Figures 3A–E**). Immunofluorescence was used to determine the anti-inflammatory efficacy of oleic acid; oleic acid dose-dependently regulated the expression of COX-2 (red fluorescence) and NF-κB p65 (green fluorescence), which were upregulated by LPS treatment (**Figure 3F**).

#### Oleic acid suppresses the expression of proinflammatory cytokines, such as TNF-α, IL-6, and IL-1β, via TLR3 and TLR4

**Figure 4:**
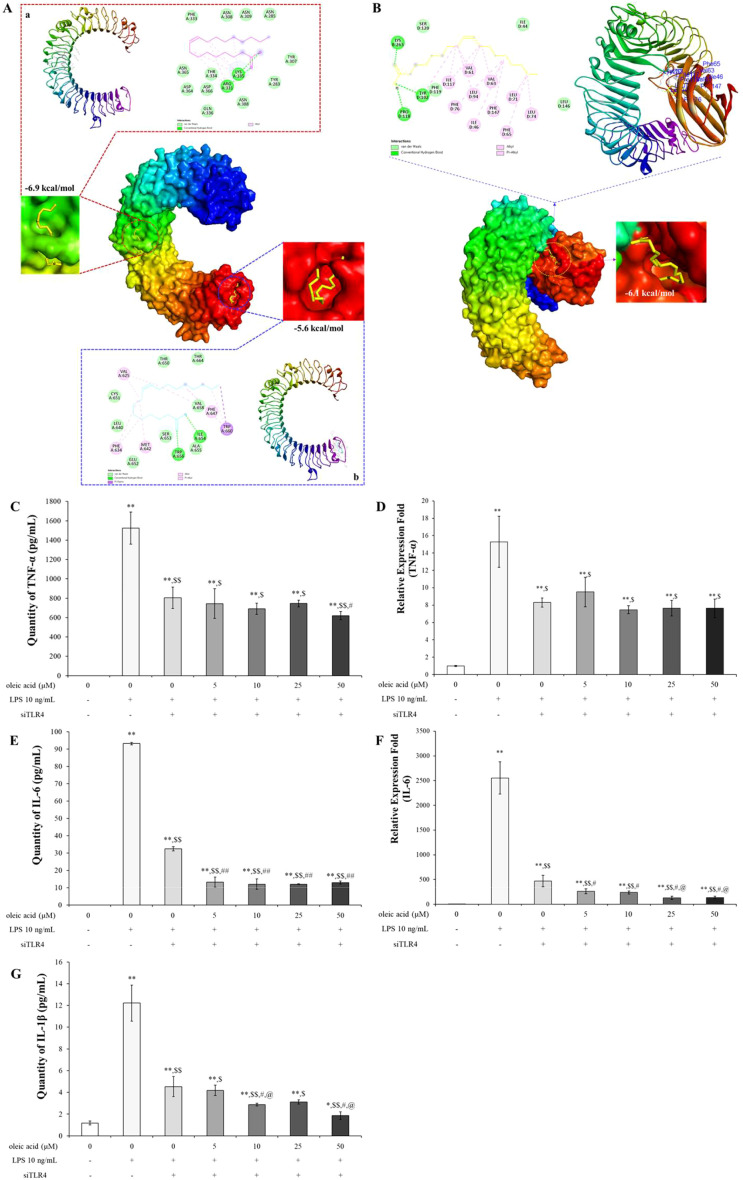
*In silico* study of oleic acid (yellow) docked into the TLR3 (TLR3 PDB ID: 1ZIW) or TLR4 (TLR4/MD-2 PDB ID: 5IJD) protein complex and the effect of oleic acid on inflammatory cytokines by TLR4 knockdown. **(A)** Oleic acid docked into the LRR 9-12 region and into the C-terminal region of TLR3. **(B)** Oleic acid docked into the TLR4/MD-2 protein complex. TLR4/MD-2 PDB ID: 5IJD. Extension of ligand and binding pose region were expressed with magenda and cyan lines border, respectively. Twenty-four hours after transfection with siTLR4, RAW 264.7 cells were incubated with oleic acid across a concentration range of 0–50 μM for 2 h, followed by LPS treatment for 2 h (n = 4). The protein and RNA levels of TNF-α **(C, D)**, IL-6 **(E, F)**, and IL-1β **(G)**, which were elevated by 10 ng/mL LPS induction, were dampened by siTLR4 transfection and similarly decreased in the oleic acid treatment group. Notably, IL-6 expression was significantly lower in the oleic acid treatment group than in the siTLR4 group. ^*^ *p* < 0.05 vs. 0 μM; ^**^ *p* < 0.001 vs. 0 μM; ^$^ *p* < 0.05 vs. 0 μM+LPS; ^$$^ *p* < 0.001 vs. 0 μM+LPS; ^#^ *p* < 0.05 vs. 0 μM+LPS+siTLR4; ^##^ *p* < 0.001 vs. 0 μM+LPS+siTLR4; ^@^ *p* < 0.05 vs. 5 μM+LPS+siTLR4; ^@@^ *p* < 0.001 vs. 5 μM+LPS+siTLR4. The results are expressed as the mean ± standard deviation.

There were 2 docking points between TLR3 and oleic acid: one was at the leucine-rich repeat (LRR) 9-12 insertion with a binding energy of -6.9 kcal/mol, which formed hydrogen bonds with ARG331 and LYS335 among other binding interactions, and the other was at the C-terminal domain, which yielded a score of -5.6 kcal/mol through interactions of hydroxyl and carbonyl groups with ILE654 and TRP656 via hydrogen bonds (**Figure 4A**). The docking point between TLR4 and oleic acid was -6.0 kcal/mol at which the carbonyl group interacted with LYS263 through a hydrogen bond, while the hydroxyl group formed hydrogen bonds with TYR102 and PRO118 (**Figure 4B**). Other hydrophobic interactions were also noted. The *in silico* findings correlated with the experimental data, corroborating the bioactivities of oleic acid on the aforementioned cytokines and receptors.

TLR4 is the primary receptor mediating immune responses to LPS, and its activation by LPS initiates several signaling pathways that lead to inflammatory responses (33). The interaction of oleic acid with TLR4 was demonstrated by its ability to inhibit the expression of cytokines following TLR4 activation through transfection. Changes in inflammatory cytokines in RAW 264.7 cells transfected with TLR4 siRNA in response to TLR4 modulation were assessed. After transfection, the expression levels of inflammatory cytokines were verified by RT−PCR and ELISA. Treatment with 10 ng/mL LPS significantly increased the protein and RNA expression of TNF-α, IL-6, and IL-1β. Cytokine expression was reduced in cells transfected with TLR4 siRNA and further decreased in the oleic acid-treated group (**Figures 4C–E, G**). Notably, both the protein and RNA levels of IL-6 were significantly lower in the oleic acid-treated group than in the siTLR4-treated group (**Figures 4E, F**), suggesting that oleic acid may interact with additional pattern recognition receptors (PRRs) beyond TLR4.

### *In vivo* study

#### Oleic acid controls representative asthmatic changes, such as increasing inflammatory cells in BALF and serum IgE and pulmonary morphological changes

**Figure 5:**
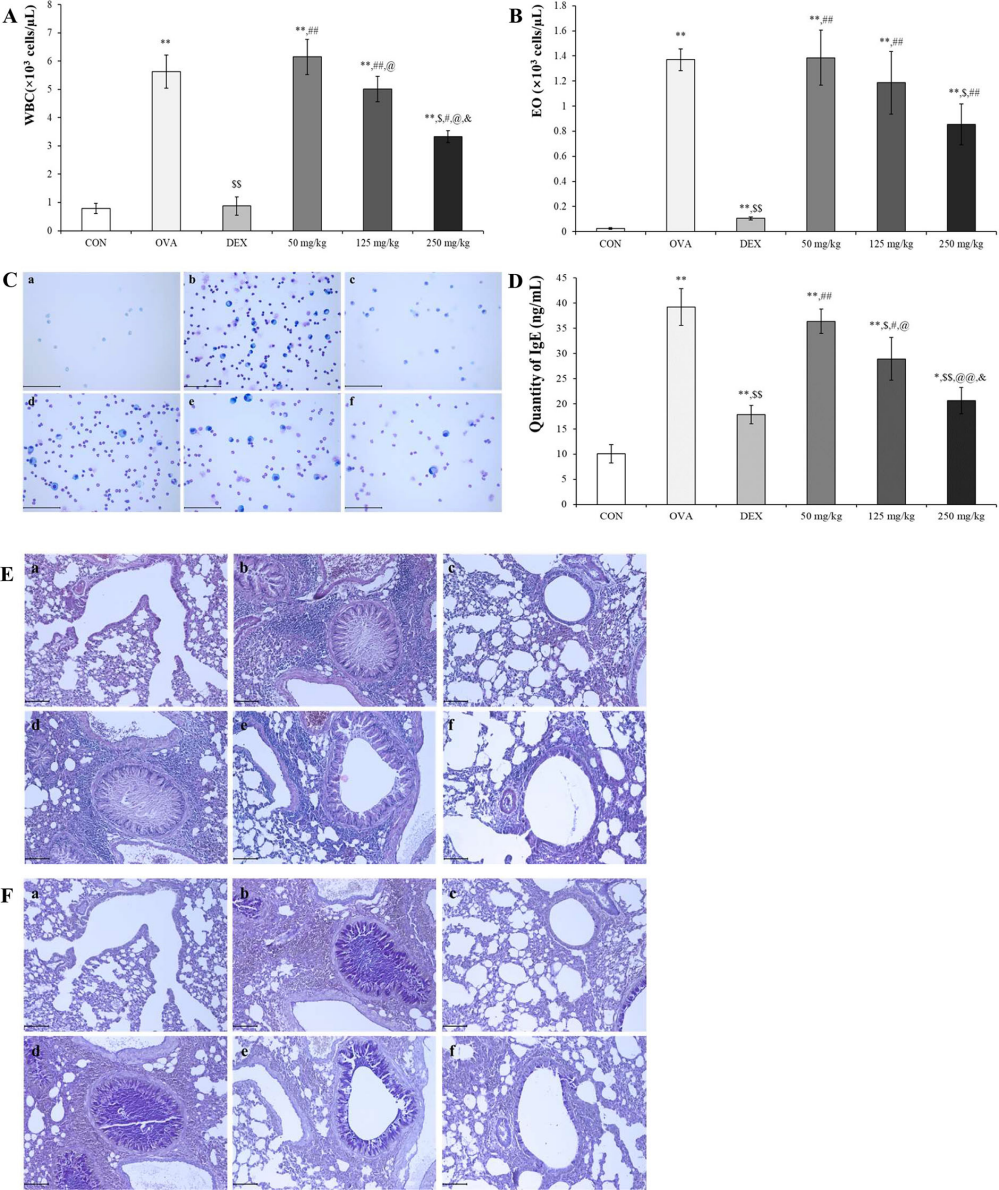
Anti-asthmatic effect of oleic acid according to BALF and serum IgE analysis and histopathological observation. The populations of **(A)** WBCs and **(B)** eosinophils (Eos) in the bronchoalveolar lavage fluid (BALF) were elevated due to OVA exposure and subsequently reduced by oleic acid treatment. Notably, oleic acid reduced the number of white blood cells and eosinophils in a concentration-dependent manner (n = 4). **(C)** Observation of inflammatory cells in BALF through Diff Quik staining showed a dose-dependent decrease with oleic acid treatment. Scale bar, 100 μm; magnification, 400×. **(D)** Serum IgE levels, which were elevated in the OVA-induced group, decreased in a dose-dependent manner with oleic acid treatment at various concentrations (n = 8). **(E)** Lung morphology, characterized by hypersecretion of mucus, proliferation of epithelial cells, and infiltration of inflammatory cells around the bronchoalveolar ducts and blood vessels, which were altered due to OVA, improved with oleic acid treatment in a dose-dependent manner. Scale bar, 100 μm; magnification, × 200. **(F)** Bronchoalveolar mucus secretion induced by OVA was reduced by treatment with varying concentrations of oleic acid (in a dose-dependent manner). Scale bar, 100 μm; magnification, × 200. a, CON; b, OVA; c, 1 mg/kg DEX; d, 50 mg/kg oleic acid; e, 125 mg/kg oleic acid; f, 250 mg/kg oleic acid. The results are expressed as the mean ± standard deviation. ^*^ *p* < 0.05 vs. CON; ^**^ *p* < 0.001 vs. CON; ^$^ *p* < 0.05 vs. OVA; ^$$^ *p* < 0.001 vs. OVA; ^#^ *p* < 0.05 vs. 1 mg/kg DEX; ^##^ *p* < 0.001 vs. 1 mg/kg DEX; ^@^ *p* < 0.05 vs. 50 mg/kg oleic acid; ^@@^ *p* < 0.001 vs. 50 mg/kg oleic acid; ^&^ *p* < 0.05 vs. 125 mg/kg oleic acid.

Compared with OVA treatment, oleic acid treatment led to a concentration-dependent reduction in the number of WBCs and eosinophils (**Figures 5A, B**). Diff Quik staining was used to determine whether oleic acid reduced the increase in inflammatory cells induced by OVA, revealing that oleic acid treatment led to a concentration-dependent reduction in inflammatory cells (**Figure 5C**). Oleic acid suppressed serum IgE levels in a dose-dependent manner, and notably, the 250 mg/kg oleic acid treatment group showed a decrease in IgE levels comparable to that of the dexamethasone treatment group (**Figure 5D**).

Histopathological analysis using H&E and PAS stains was conducted to evaluate the inhibitory effect of oleic acid on lung morphological changes induced by OVA. The OVA-treated group exhibited pronounced pathological changes, such as mucus hypersecretion, epithelial cell proliferation, and inflammatory cell infiltration. However, these changes were attenuated in a dose-dependent manner by oleic acid treatment. Specifically, the 250 mg/kg oleic acid-treated group showed improvements similar to those of the control group (**Figure 5E**). PAS staining, which reveals mucus secretion from bronchoalveolar regions, revealed a significant increase in mucus in the OVA-induced group. This secretion was mitigated in the dexamethasone-treated positive control group, and a dose-dependent decrease in mucus secretion was observed in the oleic acid-treated groups. In particular, the 250 mg/kg oleic acid-treated group exhibited a reduction in mucus secretion akin to that of the control group (**Figure 5F**).

#### Oleic acid modulates the balance between helper T cells and inactivates the NF-κB/COX-2/PGE_2_ pathway

**Figure 6:**
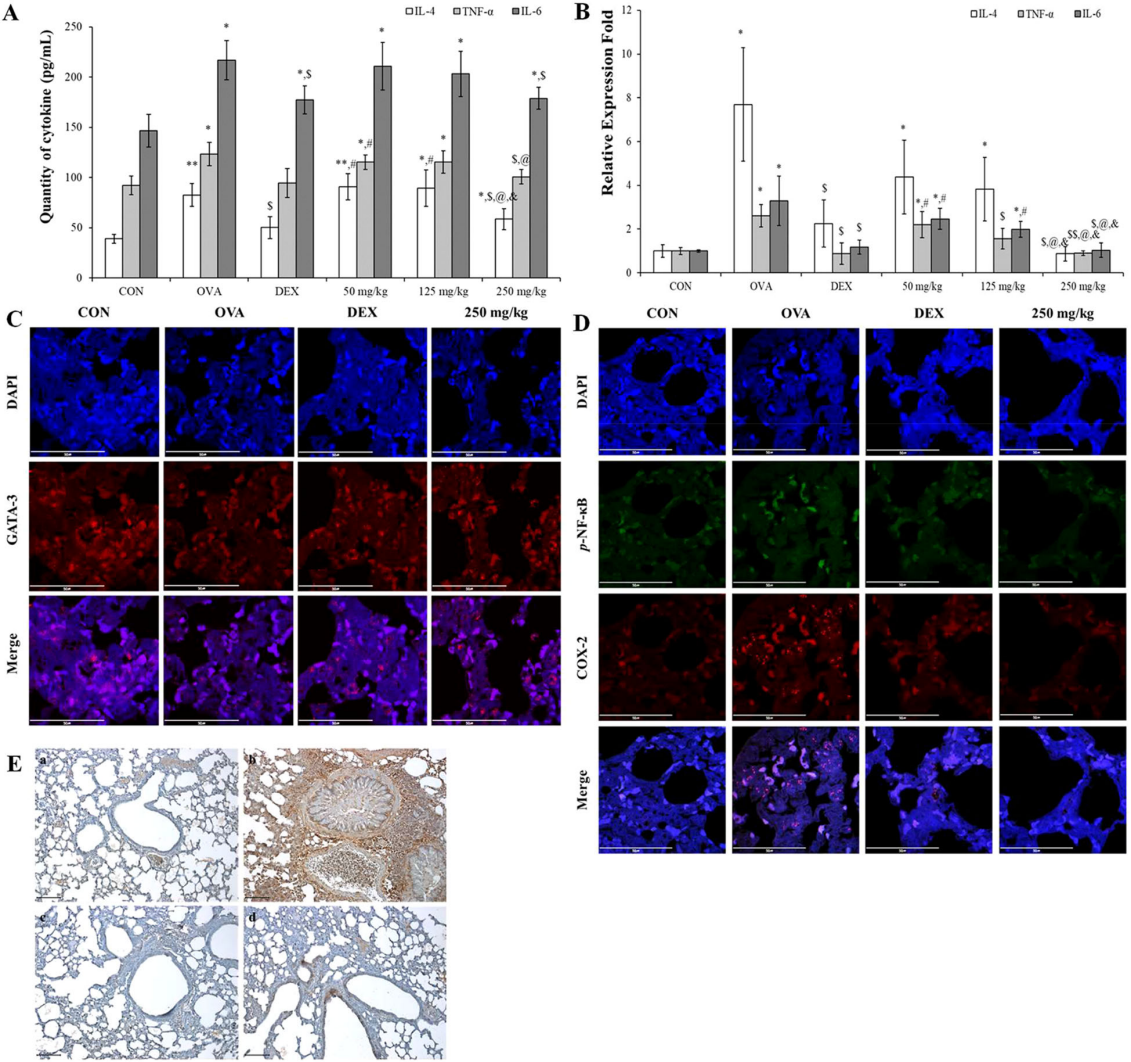
Regulation of Th2 cell and Th17 cell–related cytokines and inflammatory mechanism using oleic acid. **(A)** ELISA analysis demonstrated that oleic acid similarly reduced the protein levels of IL-4, TNF-α, and IL-6 in a dose-dependent fashion (n = 4). At 250 mg/kg, the levels were reduced to a degree akin to that of the dexamethasone treatment group. **(B)** RT−PCR analysis revealed that oleic acid reduced the cDNA levels of the Th2 cell-related cytokine IL-4 and the Th17 cell-related cytokines TNF-α and IL-6 in a dose-dependent manner (n = 4). Notably, at 250 mg/kg oleic acid, the reduction was comparable to that of the control group. **(C)** When observed by fluorescence immunostaining, compared to the merged photo in the OVA-treated group, the expression (translocation) of GATA-3 (red fluorescence) in the 250 mg/kg oleic acid-treated group was altered, similar to that in the DEX-treated group. Scale bar, 50 μm; magnification, 1000×. **(D)** Immunofluorescence indicated that oleic acid suppressed the expression of *p*-NF-κB (green fluorescence) and COX-2 (red fluorescence), which were upregulated by OVA treatment in the nucleus and cytoplasm. This suppression was similar to that observed in the DEX treatment group. Scale bar, 50 μm; magnification, ×1000. **(E)** Immunohistochemistry for PGE_2_ expression revealed that oleic acid treatment decreased the levels of PGE_2_ (brown staining), which were elevated by OVA treatment. a, CON; b, OVA; c, 1 mg/kg DEX; d, 250 mg/kg oleic acid. Scale bar, 100 μm; magnification, × 200. The results are presented as the mean ± standard deviation. ^*^ *p* < 0.05 vs. CON; ^**^ *p* < 0.001 vs. CON; ^$^ *p* < 0.05 vs. OVA; ^$$^ *p* < 0.001 vs. OVA; ^#^ *p* < 0.05 vs. 1 mg/kg DEX; ^@^ *p* < 0.05 vs. 50 mg/kg oleic acid.

To assess the impact of oleic acid on modulating the imbalance of helper T cells, we measured the expression levels of IL-4, a Th2 cell-related cytokine, and TNF-α, IL-6, and Th17 cell-related cytokines at both the RNA and protein levels. Especially TNF-α and IL-6 are very important pro-inflammatory cytokines and the change of their levels are related with not only helper T cell imbalance but also inflammation modulation. Additionally, the expression of GATA-3, a Th2 cell transcription factor, was observed via fluorescent immunostaining. In the OVA-treated group, both the cDNA and protein expression levels of IL-4, TNF-α, and IL-6 were significantly greater than those in the normal group and were lower in the DEX-treated positive control group. Treatment with varying concentrations of oleic acid resulted in a dose-dependent decrease in cytokine expression levels (**Figures 6A, B**). The translocation of GATA-3 (red fluorescence) from cytoplasm to nucleus for playing as Th2 cell transcription factor was elevated in the OVA-treated group but was reduced to translocation levels comparable to those in the normal or DEX-treated groups following oleic acid treatment (**Figure 6C**). After merging the results of DAPI and GATA-3 the level of red color in OVA was lower than those in the other groups.

Including with pro-inflammatory cytokines such as TNF-α and IL-6 the regulation of the NF-κB signaling pathway by oleic acid in lung tissue was investigated by assessing the expression of phosphorylated NF-κB, COX-2, and PGE_2_. Immunofluorescence staining revealed that the levels of phosphorylated NF-κB (green fluorescence) and COX-2 (red fluorescence), which were increased by OVA treatment, were reduced to levels similar to those in the DEX treatment group after treatment with 250 mg/kg oleic acid (**Figure 6D**). Additionally, the expression of PGE2, which is related to the NF-κB signaling pathway, was assessed via immunohistochemistry. The increase in PGE_2_ expression induced by OVA treatment decreased following treatment with 250 mg/kg oleic acid (**Figure 6E**).

#### Oleic acid modulates pulmonary epithelial cell death via downregulation of Bcl-2 expression and upregulation of Bax expression

**Figure 7:**
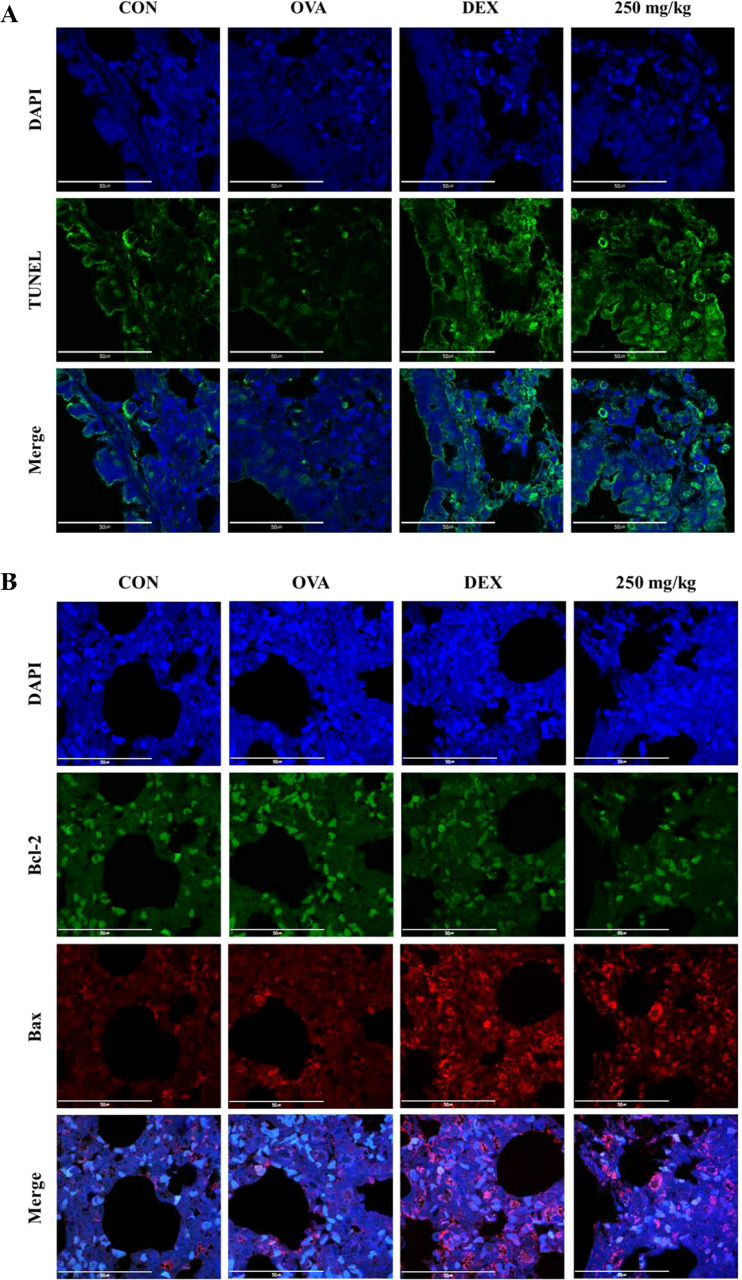
Induction of apoptosis in lung epithelial cells by oleic acid by regulating the Bcl-2 family. **(A)** TUNEL staining showed that apoptosis (green fluorescence) in lung epithelial cells, which was inhibited by OVA treatment, was significantly induced by oleic acid treatment. **(B)** Oleic acid induces apoptosis in lung epithelial cells by regulating the Bcl-2 family. Treatment with oleic acid decreased the expression of the antiapoptotic protein Bcl-2 (green fluorescence) and increased the expression of the proapoptotic protein Bax (red fluorescence). Scale bar, 50 μm; magnification, 1000×.

The TUNEL assay was used to label the free 3′-OH ends of fragmented DNA in apoptotic cells with fluorescein-dUTP via terminal deoxynucleotidyl transferase (TdT), followed by observation under a fluorescence microscope. TUNEL staining (green fluorescence, indicative of dead cells) in the airway epithelial cell region of lung tissue revealed negligible cell death in the OVA-treated group. In contrast, significant increases in TUNEL activity were observed in both the DEX-treated group and the 250 mg/kg oleic acid-treated group (**Figure 7A**).

The expression of the antiapoptotic protein Bcl-2 (green fluorescence) and the proapoptotic protein Bax (red fluorescence) was assessed using immunofluorescence staining. In the OVA-induced group, there was an increase in Bcl-2 expression and a decrease in Bax expression. Conversely, in both the dexamethasone-treated group and the 250 mg/kg oleic acid-treated group, Bcl-2 expression was downregulated, and Bax expression was upregulated (**Figure 7B**).

## Discussion

In 2022, the Global Asthma Network estimated that approximately 347 million people globally suffer from asthma. Asthma ranks among the top 10 causes of death worldwide and poses a heightened risk of fatality in children and adolescents. The etiology of asthma has not been fully elucidated; however, it is acknowledged to be a multifactorial disease, with environmental contributors such as air pollution, smoking, and allergens, as well as genetic predispositions stemming from mutations in asthma-associated genes (4). Allergic diseases are increasingly prevalent and significantly diminish quality of life (34).

The possible reasons for asthma occurrence are inflammation in the bronchioalveolar ducts (8), total T cell balance (9), and hyperplasia of respiratory system cells (10).

In mammals, 13 TLRs are known, but in humans, only 10 TLRs (TLR1 - 10) and some TLRs, such as TLR 1, TLR2, TLR4 to TLR7, and TLR10, are located in the cell membrane, but some TLRs, such as TLR3, TLR4, and TLR7 to TLR10, exist in the endoplasmic reticulum and lysosomal membrane (35). TLRs, specifically TLR3 within the endosome and TLR4 on the cell surface, recognize pathogenic invasions or external damage, triggering an immune response (16). The MAPK pathway consists of three well-known subfamilies, namely, the JNK, extracellular signal-regulated kinase (ERK), and p38MAPK subfamilies, which control NF-κB activation (18). In its inactive state, the NF-κB heterodimer, which consists of p65 and p50 proteins, is bound to NF-kappa-B inhibitor alpha (IκBα) in the cytoplasm. Upon inflammatory stimulation, IκBα is degraded, facilitating the translocation of p65 to the nucleus, which in turn activates inflammatory mediators, including COX-2 (36, 37). Moreover, the NF-κB-mediated increase in COX-2 catalyzes the conversion of arachidonic acid (AA) to the inflammatory mediator PGE_2_ (38, 39). In particular, the activation of TLR3 or TLR4 leads to the stimulation of MAPKs such as JNK and p38 MAPK, as well as the activation of NF-κB. The activation of JNK is involved in cellular responses to stress and inflammation, and p38 MAPK phosphorylation governs cell death and inflammatory responses (18). As shown in **Figure 1A** the safety of oleic acid on cells was evaluated but the statistical difference of cell viability among oleic acid dosing groups was observed 75 μM or more. As the number of live cells is deeply related to biological change such as inflammation-related cytokines the other studies were conducted under the safety level (50 μM or less). Although the statistical significance existed as the change of NO level by oleic acid was very low (**Figure 1H**) NO decrease could be caused by change of live cell number. However, anti-inflammatory effect is caused by various factors such as pro-inflammatory cytokines, anti-inflammatory cytokines, and/or reactive oxygen species and oleic acid controlled several pro-inflammatory cytokines such as IL-1β, IL-6, and TNF-α (**Figures 1C–G**). In the RAW 264.7 cell study, oleic acid suppressed the LPS-induced increases in *p*-JNK and *p*-p38 MAPK levels (**Figure 2**) and downregulated proteins related to the NF-κB signaling pathway, such as *p*-IκB and *p*-NF-κB (**Figure 3**). In BALB/c animal studies, oleic acid inhibited the OVA-induced translocation of NF-κB and synthesis of COX-2 (**Figure 6D**), thereby preventing the expression of PGE_2_ (**Figure 6E**). Consequently, oleic acid demonstrated anti-inflammatory effects related to the NF-κB pathway through the dephosphorylation of JNK and p38 MAPK. At that point, the initial mechanism of action of oleic acid involved its anti-inflammatory effect, and although there are many receptors involved in the inflammatory response, as TLRs are closely related to the NF-κB pathway via MAPK activation, the relationships between oleic acid and TLR3 and/or TLR4 were analyzed. First, an *in silico* study was performed to determine the binding affinity of oleic acid for TLR3/TLR4. Oleic acid was computationally docked to TLR3, and two binding domains pertinent to the dimerization of TLR3 and the dsRNA binding site were predicted (**Figure 4A**). Moreover, the interaction of oleic acid with TLR4 was analyzed, and a favorable docking score of -6.0 kcal/mol was observed for oleic acid bound to the NF-κB protein (**Figure 4B**). These *in silico* findings corroborate the bioactivity of oleic acid on TLR3/TLR4 receptors. In experiments with RAW 264.7 cells, cytokine production was diminished in cells transfected with TLR4 siRNA and was comparably reduced in the oleic acid-treated group. Specifically, the protein and RNA levels of IL-6 were significantly attenuated in the oleic acid treatment group relative to the siTLR4 transfection group, suggesting that the influence of oleic acid extends to TLR3 beyond TLR4 (**Figures 4E, F**).

When allergens enter the respiratory system, an overreaction of the immune system can lead to an increase in Th2 cells. These cells release proinflammatory agents that provoke asthma symptoms (9, 11). The balance between Th1 and Th2 cells suggests that an overproduction of Th2 cell-related factors represents not only an absolute increase but also a relative escalation compared to that of Th1 cell-related factors. GATA3, a crucial transcription factor for Th2 cell development, induces the expression of genes necessary for producing characteristic Th2 cell cytokines and stimulates the proliferation of these cells (40). Th2 cells generate the proinflammatory cytokines IL-4, IL-5, and IL-13, which trigger mast cell and basophil activation, leading to inflammation and airway obstruction. IL-4 enhances mucus and IgE production and stimulates mast cells, while IL-5 promotes eosinophilia. IL-13 contributes to various asthma symptoms, such as airway hyperresponsiveness, mucus production, IgE synthesis, and the activation of eosinophils and mast cells (41). The cytokines TNF-α and IL-6 are associated with Th17 cells. TNF-α acts as a proinflammatory agent and exacerbates airway hyperresponsiveness in asthma (42), while IL-6 plays a role in both inflammation and Th2 cell differentiation (43, 44). In this study, oleic acid inhibited the activation of the Th2 cell transcription factor GATA-3 (**Figure 6C**), decreased IL-4 levels (**Figures 6A, B**), and suppressed the production of dual acting cytokines that they act as pro-inflammatory and Th17-related cytokines, including TNF-α and IL-6 (**Figures 6A, B**). The downregulation of Th2-related cytokine expression by oleic acid prevented the stimulation of inflammatory cells (**Figures 5A–C**), reduced IgE production (**Figure 5D**), and inhibited mucus hypersecretion (**Figures 5E, F**). Oleic acid appears to suppress Th2 cell differentiation through the modulation of Th17-related cytokines, highlighting its efficacy as a regulator of Th2 and Th17 cells. Furthermore, oleic acid contributes to controlling eosinophils by diminishing IL-4 levels (**Figure 5B**) and modulates inflammation by downregulating TNF-α and IL-6 expression (**Figures 6A, B**), thereby exerting anti-inflammatory effects.

Bronchial epithelial cells, which act as barriers between the external environment and internal lung tissue, are instrumental in the development and progression of asthma and impact inflammation, cell death, and remodeling. The relationship between asthma and apoptosis has yielded varied results across studies due to the complex interplay of regulatory factors. Apoptosis proceeds via two primary pathways: the intrinsic pathway, which is triggered by internal cell damage or stress, and the extrinsic pathway, which is initiated by external signals such as death receptor ligands. The extrinsic pathway leads to the activation of caspases, enzymes that propagate cell death signaling. Conversely, the Bcl-2 protein family plays a critical role in the intrinsic apoptosis pathway by regulating mitochondrial membrane permeability. This family includes both antiapoptotic proteins (Bcl-2, Bcl-xL, and others) and proapoptotic proteins (Bax, Bid, Bad, Bim, and others) (45). Bcl-2 family proteins, which reside at mitochondria-endoplasmic reticulum contacts (MERCs), regulate apoptosis, cell survival, and cell migration by controlling mitochondrial Ca^2+^ dynamics. Pro-apoptotic proteins such as Bax enhance mitochondrial membrane permeability and Ca^2+^ release, inducing apoptosis. Conversely, antiapoptotic proteins such as Bcl-2 and Bcl-xL inhibit apoptosis by stabilizing mitochondrial membrane integrity and limiting Ca^2+^ release into the mitochondria (46). Some research has indicated that TGF-β promotes apoptosis in the airway epithelial cells of asthma patients through MAPK activation, contributing to the loss of these cells (47). It has also been reported that increased mitochondrial oxidative stress and decreased antioxidant enzyme activity can induce apoptosis, which may exacerbate inflammation, mucus production, and airway hyperresponsiveness (48). Conversely, other studies have suggested that the expression of PPARγ, a critical nuclear receptor involved in cellular functions, including activation, differentiation, proliferation, and death, is upregulated in the bronchial epithelial cells of asthma patients, leading to the suppression of apoptosis and the enhancement of epithelial cell proliferation. Bronchial epithelial cells have also been shown to produce both pro- and anti-inflammatory mediators in immune responses, indicating a potential shift in asthmatic patients’ bronchial epithelial cells toward an anti-inflammatory role, which might mitigate apoptosis (10). The activation of JNK (phosphorylated JNK) and p38 MAPK (phosphorylated p38 MAPK) has been associated with apoptosis through the Bcl-2 family; CdCl2 has been shown to activate the mitochondria-mediated intrinsic apoptosis pathway by reducing Bcl-2 expression and increasing Bax expression via the JNK and p38 MAPK pathways in human bronchial epithelial cells (BEAS-2B) (49). In an OVA-LPS-induced animal asthma model, anti-apoptotic effects were observed in the lung system through increased activation of p38 MAPK (*p*-p38 MAPK), inhibition of Bcl-2, and induction of Bax (21). Oleic acid dephosphorylated both JNK and p38 MAPK (**Figure 2**) and induced apoptosis by downregulating Bcl-2 expression and upregulating Bax expression in lung epithelial cells (**Figure 7**). Consequently, oleic acid prevents OVA-induced proliferation of lung epithelial cells by modulating the Bcl-2 family.

This study demonstrated that oleic acid can ameliorate the manifestation of asthma through multiple mechanisms. First, its anti-inflammatory efficacy was validated by the suppression of key components within the MAPK signaling pathways - specifically JNK and p38 MAPK - and the NF-κB signaling pathways, including IκB, NF-κB, COX-2, and PGE_2_. The modulation of inflammatory cytokines by oleic acid was further established through the knockdown of TLR4, reinforcing its association with TLR3/TLR4. Second, oleic acid was shown to rectify the imbalance between Th1 and Th2 cells by reducing the expression levels of the Th2 cell transcription factor GATA-3, as well as Th2/Th17-related cytokines such as IL-4, TNF-α, and IL-6. Finally, the promotion of apoptosis in lung epithelial cells was evidenced by the downregulation of Bcl-2 and the upregulation of Bax. Collectively, these findings suggest that oleic acid is a viable candidate for anti-asthmatic therapy.

## Data Availability

The original contributions presented in the study are included in the article/**Supplementary Material**. Further inquiries can be directed to the corresponding authors.

## Supplementary material

Supplementary Figure 1 The uncropped western blot images. **(A)** A photo of the western blot showing *p*-JNK in **Figure 2A**. **(B)** Image of the western blot showing the level of the protein encoding JNK in **Figure 2A**. **(C)** A photo of the western blot showing GAPDH in **Figure 2A**. **(D)** A photo of the western blot showing *p*-P38MAPK in **Figure 2A**. **(E)** A photo of the western membrane of P38MAPK shown in **Figure 2A**. **(F)** A photo of the western blot showing GAPDH in **Figure 2A**. **(G)** A photo of the western blot showing *p*-IκB in **Figure 3A**. **(H)** A photo of the western blot showing the presence of IκB in **Figure 3A**. **(I)** A photo of the western blot showing *p*-NFκB in **Figure 3A**. **(J)** Photo of the western blot showing the NF-κB membrane shown in **Figure 3A**. **(K)** A photo of the western blot showing GAPDH in **Figure 3A**. **(L)** A photo of the western blot image of COX-2 in **Figure 3A**. **(M)** A photo of the western membrane of PGE_2_ shown in **Figure 3A**. **(N)** A photo of the western blot showing GAPDH in **Figure 3A**.
