# Supplementary figures and images for: Oleic acid attenuates asthma pathogenesis via Th1/Th2 immune cell modulation, TLR3/4-NF-κB-related inflammation suppression, and intrinsic apoptotic pathway induction
Source: Front Immunol. 2024 Oct 3;15:1429591. doi: 10.3389/fimmu.2024.1429591 (PMC11484255; doi:10.3389/fimmu.2024.1429591)

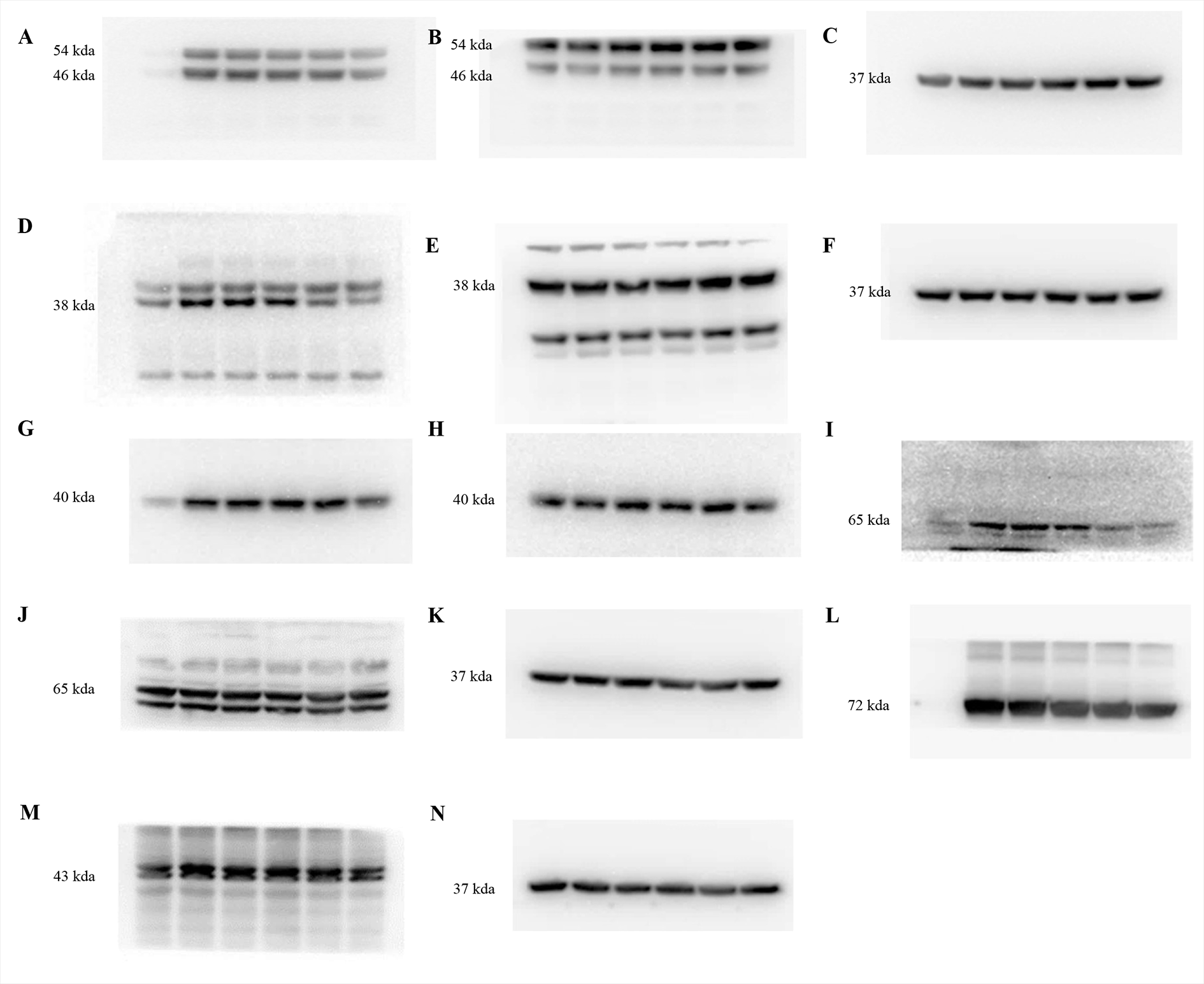

Supplement: Supplementary Figure 1 — The uncropped western blot images. (A) A photo of the western blot showing p-JNK in Figure 2A . (B) Image of the western blot showing the level of the protein encoding JNK in Figure 2A . (C) A photo of the western blot showing GAPDH in Figure 2A . (D) A photo of the western blot showing p-P38MAPK in Figure 2A . (E) A photo of the western membrane of P38MAPK shown in Figure 2A . (F) A photo of the western blot showing GAPDH in Figure 2A . (G) A photo of the western blot showing p-IκB in Figure 3A . (H) A photo of the western blot showing the presence of IκB in Figure 3A . (I) A photo of the western blot showing p-NFκB in Figure 3A . (J) Photo of the western blot showing the NF-κB membrane shown in Figure 3A . (K) A photo of the western blot showing GAPDH in Figure 3A . (L) A photo of the western blot image of COX-2 in Figure 3A . (M) A photo of the western membrane of PGE2 shown in Figure 3A . (N) A photo of the western blot showing GAPDH in Figure 3A . [file Image1.tif]
